# Oxidative stress and inflammation: determinants of anthracycline cardiotoxicity and possible therapeutic targets

**DOI:** 10.1007/s10741-020-10063-9

**Published:** 2020-12-15

**Authors:** Iacopo Fabiani, Alberto Aimo, Chrysanthos Grigoratos, Vincenzo Castiglione, Francesco Gentile, Luigi F Saccaro, Chiara Arzilli, Daniela Cardinale, Claudio Passino, Michele Emdin

**Affiliations:** 1 Cardiology Division, Fondazione Toscana Gabriele Monasterio, Pisa, Italy; 2 Institute of Life Sciences, Scuola Superiore Sant’Anna, Pisa, Italy; 3 University Hospital of Pisa, Pisa, Italy; 4 Cardioncology Unit, European Institute of Oncology, IRCCS, Milan, Italy

**Keywords:** Anthracyclines, Cardiotoxicity, Oxidative stress, Inflammation

## Abstract

Chemotherapy with anthracycline-based regimens remains a cornerstone of treatment of many solid and blood tumors but is associated with a significant risk of cardiotoxicity, which can manifest as asymptomatic left ventricular dysfunction or overt heart failure. These effects are typically dose-dependent and cumulative and may require appropriate screening strategies and cardioprotective therapies in order to minimize changes to anticancer regimens or even their discontinuation. Our current understanding of cardiac damage by anthracyclines includes a central role of oxidative stress and inflammation. The identification of these processes through circulating biomarkers or imaging techniques might then be helpful for early diagnosis and risk stratification. Furthermore, therapeutic strategies relieving oxidative stress and inflammation hold promise to prevent heart failure development or at least to mitigate cardiac damage, although further evidence is needed on their efficacy, either alone or as part of combination therapies with neurohormonal antagonists, which are the current adopted standard.

Cardiotoxicity manifesting as asymptomatic left ventricular (LV) dysfunction or overt heart failure (HF) is a major adverse effect of chemotherapy for cancer [1, 2]. In a recent analysis from the CARDIOTOX Registry, which prospectively evaluated 865 patients undergoing anticancer therapy associated with a moderate to high risk of cardiotoxicity, as many as 38% of patients displayed overt cardiotoxicity over a 24-month follow-up, with severe cardiac disease occurring in 3% [3]. These last patients had a mortality rate of 23 deaths per 100 patients-year.

Introduced in the 1960s, anthracyclines (ANT) are among the most potent and prescribed chemotherapeutics for the treatment of haematological and solid tumors. The most used drug of this class is doxorubicin (DOX), also known as adriamycin, with several other molecules used, including daunorubicin, epirubicin, and valrubicin. LV dysfunction and HF are well-established, typically dose-dependent, and cumulative adverse effects of ANT, and may require changes to anticancer regimens or even their discontinuation[1]*.* HF has a 5% incidence in patients receiving a cumulative DOX dose of 400 mg/m^2^, increasing to 48% with a cumulative dose of 700 mg/m^2^, but even low doses (such as 100 mg/m^2^) may cause cardiac dysfunction [4]. In addition to the cumulative dose, major risk factors include the administration schedule, other cardiotoxic therapies (such as trastuzumab or paclitaxel), mediastinal irradiation, and history of heart disease [1]. Cardiotoxic reactions to ANT may be acute (i.e., occurring during drug administration), subacute, or chronic. However, recent findings challenge this old classification, suggesting that anthracycline-induced cardiotoxicity is potentially a continuous phenomenon, starting with myocardial cell injury, followed by progressive functional decline, progressively leading to overt HF [5, 6]. Patient susceptibility to ANT is highly variable, with many patients tolerating therapeutic dose of ANT without long-term complications, while others showing ANT-dependent cardiotoxicity even after the first dose. Some patients may develop cardiotoxicity at a total cumulative dose of ANT corresponding to 300 mg/m^2^, or even lower, while others have no significant cardiac alterations, despite being exposed to doses up to 1000 mg/m^2^ [4].

Therapies for neurohormonal antagonism are currently recommended for patients with a normal LV ejection fraction (LVEF) and cardiovascular risk factors scheduled to undergo therapy with cardiotoxic agents, although this evidence derives from a limited number of studies [7, 8].

The development of cardiotoxicity by ANT may be partially explained by the induction of oxidative stress and inflammatory responses [4]. A deeper knowledge on these processes might disclose new possibilities for early detection of cardiac damage (through circulating biomarkers or imaging findings related to inflammation) and offer new therapeutic strategies, complementary to the use of neurohormonal antagonists (Fig. 1).

**Fig. 1:**
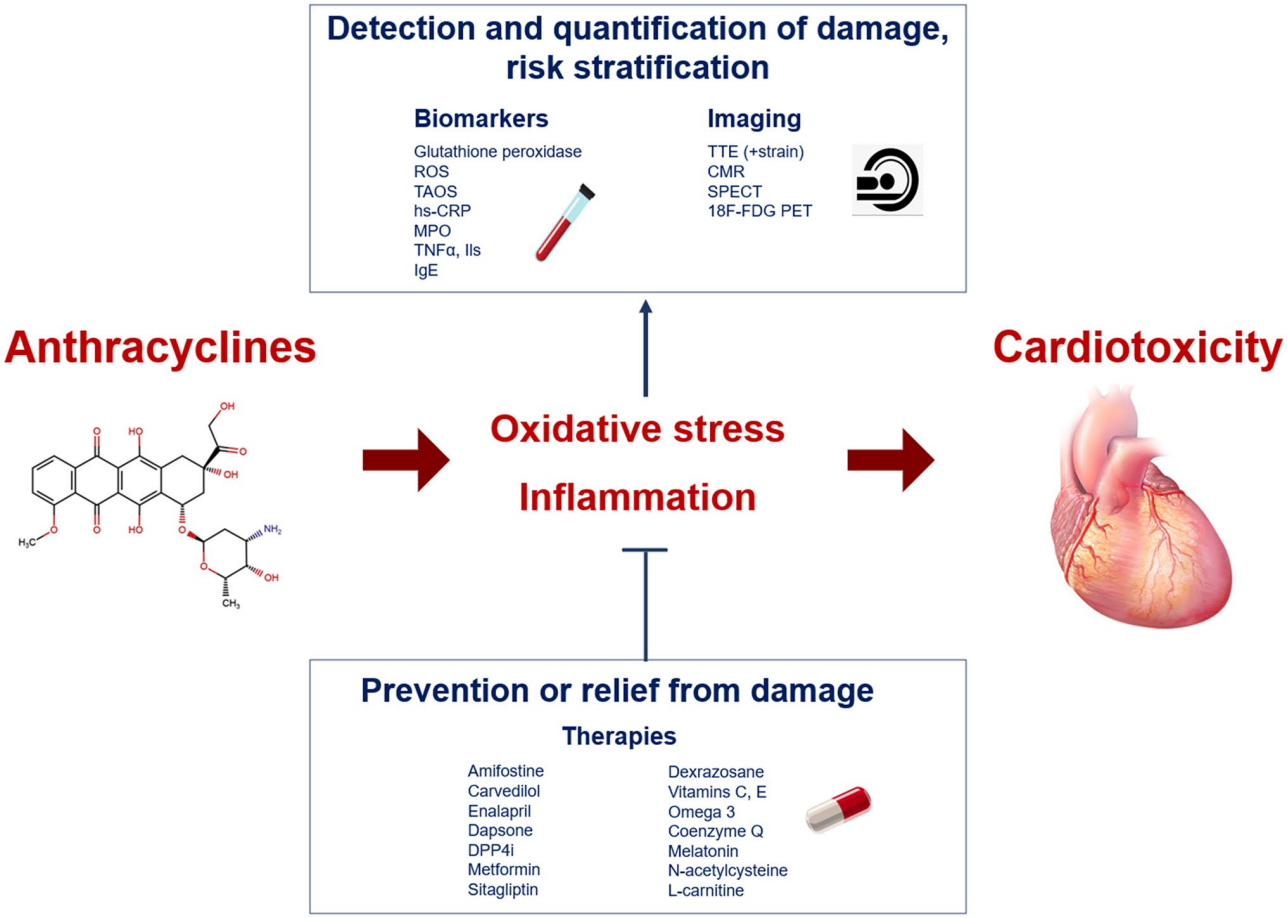
Central illustration. Oxidative stress and inflammation in anthracycline cardiotoxicity.^18^F-FDG PET, ^18^F-fluoro-D-glucose positron emission tomography, CMR cardiovascular magnetic resonance, DPP4i dipeptidyl-peptidase 4 inhibitor, hs-CRP high-sensitivity C-reactive protein, IgE immunoglobulin E, IL interleukin, MPO myeloperoxidase, ROS reactive oxygen species, SPECT single-photon emission computed tomography, TAOS total antioxidant status, TNFα tumor necrosis factor alpha, TTE transthoracic echocardiogram

For the present review, we performed a search on PubMed and EMBASE in May 2020 with the following terms: “anthracycline” OR “doxorubicin” AND “cardiotoxicity” OR “cardiac damage” OR “heart failure” OR “cardiac failure.” The reference list of relevant articles was also searched; only articles published in English were included. Given the design of this work as a narrative review, no formal criteria for study selection or appraisal were enforced.

## Oxidative stress and inflammation as possible contributors to cardiac damage

ANT cardiotoxicity is believed to derive from DNA damage, inhibition of protein synthesis and mitochondrial biogenesis, induction of apoptosis, inflammation and generation of reactive oxygen species (ROS). The myocardium has a high mitochondrial density [9], and the propensity of ANT to accumulate in the mitochondria promotes their retention in cardiomyocytes. Here, ANT metabolites interfere with the translation of iron sequestrating proteins, thus increasing intracellular free iron, which leads to the generation of highly reactive iron-ANT complexes, capable of starting redox cycling and promoting excessive autophagy [10–12]. ANT may also easily intercalate into mitochondrial DNA, because of its circular and covalently closed structure, further promoting mitochondrial respiratory chain dysfunction [13–15]. Finally, ANT may directly activate ROS-producing enzymes. Notably, ROS generation is usually associated with the production of equally dangerous reactive nitrogen species (RNS). Indeed, ANT have been found to increase the activity of inducible nitric oxide synthase (NOS), which can foster RNS generation [16, 17]. Once antioxidant defences have been overwhelmed, ROS may react with lipids, proteins, and DNA, leading to the disruption of mitochondrial integrity and function that may ultimately cause cell death [18]. ROS-dependent cellular damages may induce inflammation, which is a crucial mechanism in HF development. Interestingly, phenylalanine-butyramide (a synthetic derivative of the short-chain fatty acid butyric acid) has been recently shown to reduce oxidative stress and mithocondrial dysfunction in a murine model of ANT cardiotoxicity [19].

ANT therapy is also associated with induction of nuclear factor-κB (NFkB) and tumor necrosis factor alpha (TNF-α). Furthermore, several Toll-like receptors (TLRs), a class of pattern recognition receptors involved in innate immune response initiation and induction of cytokines secretion, are activated and promote the activation of adaptive immunity cells (for instance, TLR4 activates T helper-1 cells) [4, 20]. Furthermore, through transcription factor modulation, ANT increase NLRP3 expression, thus stimulating the release of the proinflammatory interleukins (IL)-1β and IL-6 [21]. Additionally, ANT-dependent inhibition of cyclooxygenase and lipoxygenases reduces cardiac availability of anti-inflammatory mediators (such as prostacyclins), promoting cardiac damage [22, 23].

Cardiomyocyte damage and death further amplify the inflammatory cascade and oxidative stress described above, involving cytokines, cardiomyocytes, immune, and endothelial cells. This vicious cycle induces the functional and structural alterations (cardiac hypertrophy, fibrosis, electrical abnormalities) observed in HF.

## Biomarkers of oxidative stress and inflammation

Serum biomarkers have been studied as tools for risk stratification before ANT therapy, early detection of subclinical cardiac damage during treatment, or identification of late effects. The majority of studies have focused on cardiac troponins and natriuretic peptides (NPs), which have been recommended as tools for therapy monitoring [1].

Evidence on biomarkers of oxidative stress and inflammation is sparse and mostly derives from single-center studies. The increase in serum *ROS* (determined by measuring the radical species produced by a specific reaction, which are directly proportional to the quantity of lipid peroxides) and the decrease in *glutathione peroxidase* displayed a correlation with the reduction in strain rate peak, and serum ROS levels were the only independent predictive variable of systolic dysfunction at multiple regression analysis [24]. In a phase II trial on 49 cancer patients receiving epirubicin and either telmistartan or placebo, telmisartan was able prevent ANT-induced increase in serum IL-6 and ROS levels [25]. *Total antioxidant status* (TAOS) represents the sum of all measurable antioxidant within plasma and body fluids. In a study on 29 acute leukemia children on ANT, TAOS levels decreased in parallel with the cumulative ANT dose [26].

*C-reactive protein* (CRP) is a nonspecific marker of inflammation produced by the hepatocytes in response to IL-6 released from macrophages and T cells. Several studies reported a significant elevation of CRP [27] and high-sensitivity CRP (hs-CRP) [28–31] following ANT-based chemotherapy, but only one study demonstrated a modest association between hs-CRP increase and echocardiographic evidence of cardiotoxicity [31], and therapies counteracting ANT-related cardiotoxicity do not seem to alter hs-CRP levels [32].

*Myeloperoxidase* (MPO) is a proinflammatory enzyme expressed by neutrophils and involved in ROS production and lipid peroxidation. In one study on 78 cancer patients receiving doxorubicin and trastuzumab, a transient increase in MPO levels following chemotherapy predicted subsequent development of LVEF decline; among several biomarkers, the combination MPO and troponin I identified a subgroup of patients at higher risk of cardiotoxicity [30]. A following study from the same group confirmed the predictive value of MPO for ANT cardiotoxicity [31].

Inflammatory mediators *TNFα* and *ILs* have not emerged as robust biomarkers of ANT cardiotoxicity. Serum TNF and soluble TNF receptors I and II levels do not seem to change in response to ANT-based chemotherapy [24, 33, 34]. Similar results have been reported for IL-1β [24]. Conversely, IL-6 levels increased significantly following epirubicin administration and correlated with reduction of peak strain rate at echocardiography, a marker of early systolic dysfunction [24].

Sawaya et al. [35] and Armenian et al. [36] evaluated the predictive role of soluble suppression of tumorigenesis 2 (*sST2*), a biomarker released mainly by extracardiac tissues in response to inflammatory and fibrotic stimuli, for early and late cardiotoxicity in cancer survivors treated with ANT-based regimens. Both studies showed that sST2 levels were slightly above normal range at baseline, but did not change significantly during the follow-up and did not correlate with the evolution of echocardiographic parameters. A recent study reported a significant increase in sST2 following ANT administration to breast cancer patients, although no difference in sST2 were observed between patients who developed a decline in LVEF and those remaining clinically stable [37].

Multiomics approaches have tried to trace the molecular fingerprint of ANT cardiotoxicity in order to identify novel biomarkers able to predict this complication. For example, a proteomic profiling approach showed that higher baseline *immunoglobulin E *(*IgE*) levels were associated with a lower risk of chemotherapy-induced cardiotoxicity, possibly because of a lower degree of activation of the Th1 response [38].

## Imaging findings related to inflammation

Several imaging techniques allow to assess the presence and extent of inflammation and to monitor its evolution over time (Table 1). In the acute phase, imaging findings may vary from a subclinical injury with preserved LVEF [5] up to extensive involvement and cardiogenic shock [39]. Echocardiography represents the first-line imaging technique, but subtle systolic abnormalities may not be recognized during the early phases of the disease. In the setting of ANT cardiotoxicity, strain echocardiography may not only detect subtle alterations but also predict subsequent myocardial dysfunction; therefore, its use in everyday clinical practice is recommended by current guidelines [40].

**Table 1:** Imaging techniques to assess myocardial inflammation and its consequences in patients receiving anthracyclines (ANTs)

Acute/chronic phase, *roles of imaging*	Imaging technique	Main findings in ANT cardiotoxicity
Acute phase: - Early detection of cardiac damage - Prognostic stratification	(2D/3D) TTE	LV/RV regional/global systolic dysfunction; diastolic dysfunction
	(2D/3D) strain TTE	Subclinical LV/RV systolic dysfunction
	CMR	LV/RV regional/global systolic dysfunction; LV/RV hyperintensity in T2w sequences; LGE; increase in regional/global T1 and T2 mapping values
	SPECT	LVEF decrease
	^18^F-FDG PET	Increased glucose uptake
Chronic phase: - Detection of cardiac damage - Follow-up of known cardiomyopathy	TTE	LV/RV regional/global systolic dysfunction; diastolic dysfunction
	CMR	LV/RV regional/global systolic dysfunction; LGE
	SPECT	LVEF decrease

*2D/3D* two-/three-dimensional, ^*18*^*F-FDG PET,* ^18^F-fluoro-D-glucose positron emission tomography, *CMR* cardiac magnetic resonance, *LGE* late gadolinium enhancement, *LV/RV* left/right ventricle, *TTE* transthoracic echocardiography

The main limitation of echocardiography is that it can assess myocardial damage only through its impact on systolic function. Conversely, cardiac magnetic resonance (CMR) allows not only to reproducibly estimate biventricular volumes and systolic function but also to assess the presence, extent, and pattern of distribution of myocardial edema, fat, and fibrosis [41–43]. T2-weighted fast-spin-echo sequence is a robust technique in inflammation imaging, allowing to noninvasively detect with a millimetric spatial resolution the presence of areas of myocardial inflammation in various inflammatory cardiomyopathies including early-phase ANT cardiotoxicity [28]. More recently, novel parametric mapping techniques such as T1 and T2 mapping have shown their potential in the early identification of ANT cardiotoxicity in both preclinical [44] and clinical settings [45].

Single-photon emission computed tomography (SPECT) has been used to assess LV geometry and function in patients receiving ANT [46] but is performed very rarely because of the radiation exposure of patients requiring follow-up examinations, and the low agreement between SPECT and CMR estimates of LVEF [47]. ^18^F-Fluoro-D-glucose positron emission tomography (^18^F-FDG PET) has the unique ability to characterize myocardial metabolism [48], which may be helpful for early detection of cardiotoxicity as increased glucose uptake [49]. The role of ^18^F-FDG PET is currently limited to the research setting.

Following the acute phase, serial CMR examinations may document the evolution of myocardial inflammation and its sequelae. Myocardial scar can be detected as late gadolinium enhancement (LGE) in post-contrast T1 gradient echo sequences. Various LGE patterns and estimates of LGE prevalence have been reported in ANT-related cardiotoxicity [50, 51], likely because of the small study size, and their heterogeneous enrolment criteria and treatment regimens. Both reparative fibrosis and interstitial fibrosis have been described. In children with subclinical ANT cardiotoxicity, T1 mapping showed an expansion of extracellular spaces, correlating with cumulative ANT dose and exercise capacity [52]. In a cohort of adult cancer survivors, myocardial T1 elevation occurred independently of the underlying neoplasm or cardiac comorbidities, and could then be attributed to the cardiotoxic effect of chemotherapy [53].

## Therapeutic approaches

Several treatments targeting oxidative stress or inflammation have been evaluated as possible approaches to ANT-related cardiotoxicity (Table 2). On the other hand, the identification of a less cardiotoxic analogous of ANT has proven challenging [54]; these approaches will not be discussed here.

**Table 2:** Therapeutic approaches targeting oxidative stress and inflammation for the prevention of anthracycline (ANT)-related cardiotoxicity: evidence from preclinical studies and clinical trials

Antioxidant agents				
First Author, year (ref.)	Molecule	Study design	Population	Main results
Macedo et al., 2019 [56]	Dexrazoxane	Meta-analysis	Patients with BC from 9 clinical trials (*n* = 2177)	Reduction in cardiac events and HF incidence; no effects on the efficacy of anticancer therapy
Akolkar et al., 2017 [61]	Vitamin C	In vitro study	Rat CMs	Inhibition of pro-oxidant and pro-inflammatory cascade
Akolkar et al., 2017 [62]	Vitamin C	Animal study	Rats receiving ANT	Inhibition of pro-oxidant and pro-inflammatory cascade, lower rates of cardiac damage, systolic and diastolic dysfunction
Berthiaume et al., 2005 [63]	Vitamin E	Animal study	Rats receiving ANT	No prevention of mitochondrial dysfunction and histological changes in the heart
Carbone et al., 2012 [64]	Omega 3 PUFA	Animal study	Sheep receiving ANT	Exacerbation of ANT cardiotoxicity
Iarussi et al., 1994 [68]	Coenzyme-Q	Clinical study (not RCT)	Patients with ALL (*n* = 20)	Reduction in LV systolic dysfunction and wall motion abnormalities
Chen et al., 2016 [69]	Coenzyme-Q	Animal study	Rats receiving ANT	Reduction in histological changes in the heart
Najafi et al., 2019 [70]	Melatonin	Systematic review of pre-clinical studies	28 studies (mostly on mice/rats)	Decreased mortality, body weight and heart weight, ascites; reduction of histological changes in the heart
Myers et al., 1983 [71]	N-acetylcysteine	RCT	Patients with solid tumors (*n* = 54)	No difference in HF incidence
Waldner et al., 2006 [72]	L-carnitine	RCT	Patients with NHL (*n* = 40)	No difference in HF incidence
Gallegos-Castorena et al., 2007 [73]	Amifostine	Clinical study (not RCT)	Patients with osteosarcoma (*n* = 28)	No difference in HF incidence
Kheiri et al., 2018 [77]	Carvedilol	Meta-analysis	Patients with solid or blood cancer (*n* = 633)	Reduction in HF incidence and of absolute LVEF decrease
Bosch et al., 2013 [78]	Carvedilol/enalapril	RCT	Patients with blood cancer (*n* = 90)	Preservation of LV systolic function
Hiona et al., 2011 [81]	Enalapril	Animal study	Rats receiving ANT	Preservation of LV systolic function and mitochondrial respiratory efficacy
Anti-inflammatory agents				
Reference	Molecule	Study design	Population	Main results
Sheibani et al., 2020 [86]	Dapsone	Animal study	Rats receiving ANT	Reduction in cardiac levels of prooxidant and proinflammatory factors; preservation of cardiac function
Sheta et al., 2016 [83]	Metformin or sitagliptin	Animal study	Rats receiving ANT	Blunting of inflammatory pathways and preservation of cardiac function (+ + sitagliptin)
Peng et al., 2019 [84]	Teneligliptin	In vitro study	Rat and human CMs	Reduction in CM damage, apoptosis, and proinflammatory cytokines
Seicean et al., 2012 [87]	Statins	Retrospective study	Patients with BC (*n* = 628)	Reduction in HF incidence and cardiac mortality

*ALL* acute lymphoblastic leukemia, *BC* breast cancer, *CM* cardiomyocyte, *HF* heart failure, *IL-1* interleukin-1, *LV* left ventricle, *LVEF* left ventricular ejection fraction, *NHL* non-Hodgkin lymphoma, *PUFA* polyunsaturated fatty acids, *RCT* randomized clinical trial, *TNFα* tumor necrosis factor alpha

### Antioxidant agents

*Dexrazoxane* (DEX) is the only drug approved for the primary prevention of ANT cardiotoxicity. DEX is an iron-chelator that reduces ROS production by impairing the formation of iron-ANT complexes; this molecule also inhibits cardiomyocyte apoptosis by blocking ANT binding to topoisomerase IIβ [55]. A significant reduction in cardiac events and HF incidence in a cohort of 2177 patients with breast cancer exposed to ANT and treated with DEX, without any reduction in the antineoplastic efficacy of the chemotherapy regimens, has been reported [56]. DEX is currently approved in Europe for adults with advanced metastatic breast cancer who have received a cumulative dose ≥ 300 mg/m^2^ of doxorubicin or ≥ 540 mg/m^2^ of epirubicin and would benefit from continued ANT-based therapy [1]. Despite some concerns of short- and long-term risks (e.g., myelosuppression, acute myeloid leukemia), studies have shown a generally safe profile even in younger patients [57–59]. Therefore, a recent opening has been made for the use of DEX in patients under 18 years requiring high ANT doses (https://www.ema.europa.eu/en/medicines/human/referrals/cardioxane).

Other antioxidants have been tested [60]. Similar to DEX, these molecules are believed to reduce ROS-related cardiac damage and, at least partially, inhibit cardiomyocyte apoptosis. One of these molecules is *vitamin C*, which was reported to mitigate DOX-induced oxidative and nitrosative stress and apoptosis in isolated cardiomyocytes [61], and to blunt inflammatory activation, improve systolic and diastolic function, and survival in rats exposed to DOX [62]. Similarly, *vitamin E* relieved oxidative stress in rats, but was not sufficient to protect cardiac mitochondrial membranes from DOX toxicity [63].

The antioxidant *omega-3 polyunsaturated fatty acids *(*PUFA*) failed to afford cardiac protection to sheep receiving ANT [64], and an open-label, phase II clinical trial showed no significant adverse effects and preserved efficacy of chemotherapy in patients receiving ANT [65].

*Coenzyme Q10* is a fat-soluble electron carrier in mitochondria and a coenzyme in different energetic pathways [66]. A significant decrease in myocardial content of coenzyme Q10 has been observed in ANT-related cardiomyopathy [67]. Supplementation of this molecule has been tested in both pre-clinical studies [68, 69] and small clinical trials [68, 69], whose results do not allow any definite conclusion.

*Melatonin* is a hormone with antioxidant effect that can be supplemented with limited adverse effects. Several preclinical studies have investigated the role of melatonin in ANT-related cardiotoxicity. Based on a recent meta-analysis of 28 studies, melatonin administration appeared safe and associated with reduced cardiac damage and mortality [70]. *N-acetylcysteine* has a well-known antioxidant efficacy but did not reduce HF occurrence in the only randomized controlled trial (RCT) on patients with solid cancers treated with ANT [71]. The antioxidant molecules *l-carnitine* and *amifostine* seem to not provide any benefit to human patients treated with ANT [72, 73].

*Carvedilol*, a β-1, β-2, and α-1 adrenergic receptor blocker, has an antioxidant action deriving from the inhibition of the complex 1 of the respiratory chain [74] and reduces lipid peroxidation [75, 76]. In a meta-analysis including 8 trials and a total of 633 patients, co-treatment with carvedilol was associated with a lower rate of both HF occurrence and absolute decrease in LV ejection fraction, although the reliability of this conclusion is limited by the high degree of heterogeneity in the study populations and endpoints [77]. The OVERCOME (preventiOn of left Ventricular dysfunction with Enalapril and caRvedilol in patients submitted to intensive ChemOtherapy for the treatment of Malignant hEmopathies) study was a small (*n* = 90 patients) trial showing a protective role of the combination of carvedilol and the angiotensin-converting enzyme inhibitor (ACEi) enalapril in patients with blood neoplasms, 40% of whom receiving ANT [78]. Several other studies reported the positive effects of *ACEi* and *angiotensin receptor blockers* in preventing ANT cardiotoxicity, because of their anti-neurohormonal, vasodilating, and possibly antioxidant and anti-inflammatory actions [79–82].

### Anti-inflammatory agents

Secondary anti-inflammatory and potential antioxidant properties have been attributed to some molecules routinely used in different clinical settings. The anti-diabetic drugs *dipeptidyl-peptidase 4 inhibitors* (DPP4i) seem to inhibit inflammatory and pro-oxidant pathways. In a rat model of DOX-toxicity, the DPP4i *sitagliptin* antagonized the inflammatory cascade involved in ANT cardiotoxicity, reducing cell damage, apoptosis, and cytokine levels [83]. A class-dependent effect of DPP4i might be postulated given the efficacy of another molecule (*teneligliptin*) in a recent in vitro study [84]. Similarly, the inhibitors of the sodium glucose co-transporter 2 *empagliflozin* and *dapagliflozin* appear to reduce ANT-dependent inflammation and cardiotoxicity in vitro and in vivo [85]. Furthermore, the antibiotic *dapsone* was tested in rats that had received DOX, leading to a reduction in heart levels of oxidant factors and pro-inflammatory cytokines and a significant amelioration of electrocardiographic and electrophysiological parameters, heart contractility, and biomarkers concentrations, as well as positive effects at the histopathological level [86]. Finally, the potent anti-inflammatory and anti-oxidant effects of *statins* might explain the beneficial effects of these molecules in preventing incident HF in a cohort of 628 newly diagnosed breast cancer patients receiving ANT [87]. An RCT is underway to verify the efficacy of atorvastatin in this clinical setting (NCT01988571).

## Conclusions and future perspectives

Chemotherapy with ANT-based regimens remains a cornerstone of treatment of many solid and blood tumors, but is associated with a nonnegligible risk of cardiotoxicity, which may lead over time to clinically manifest HF. Our current understanding of cardiac damage by ANT includes a central role of oxidative stress and inflammation. Detection of these processes through circulating biomarkers or imaging techniques might then be helpful for early diagnosis and risk stratification. Furthermore, therapeutic strategies relieving oxidative stress and inflammation hold some promise to prevent HF development or at least mitigate cardiac damage, although further evidence is needed on the efficacy of these drugs, either alone or as part of combination therapies with therapies for neurohormonal antagonism.

## Key points

- Anthracyclines are potent and common chemotherapeutics for hematological and solid tumors but are burdened with a serious risk of cardiotoxicity, which may be partly mediated by inflammation and oxidative stress.
- Several serum biomarkers related to inflammation and oxidative stress (e.g., CRP, myeloperoxidase, glutathione peroxidase) have been proposed for risk stratification and detection of anthracyclines cardiotoxicity, but evidence is still lacking.
- Anthracycline cardiotoxicity can be monitored with imaging techniques, e.g., echocardiography, computed or positron emission tomography, or cardiac magnetic resonance that can document the evolution of myocardial inflammation and its sequelae.
- Apart from dexrazoxane, which reduces ROS production, no other drug is approved for the primary prevention of ANT cardiotoxicity, but several anti-inflammatory and antioxidant agents are being investigated.
